# Supplementary material for: Fungal oxylipins direct programmed developmental switches in filamentous fungi
Source: Nat Commun. 2020 Oct 14;11:5158. doi: 10.1038/s41467-020-18999-0 (PMC7557911; doi:10.1038/s41467-020-18999-0)
Supplement: Supplementary file 1 — Supplementary Information [file 41467_2020_18999_MOESM1_ESM.pdf]

Supplementary Information

Fungal oxylipins direct programmed developmental switches in filamentous fungi

Niu et al.

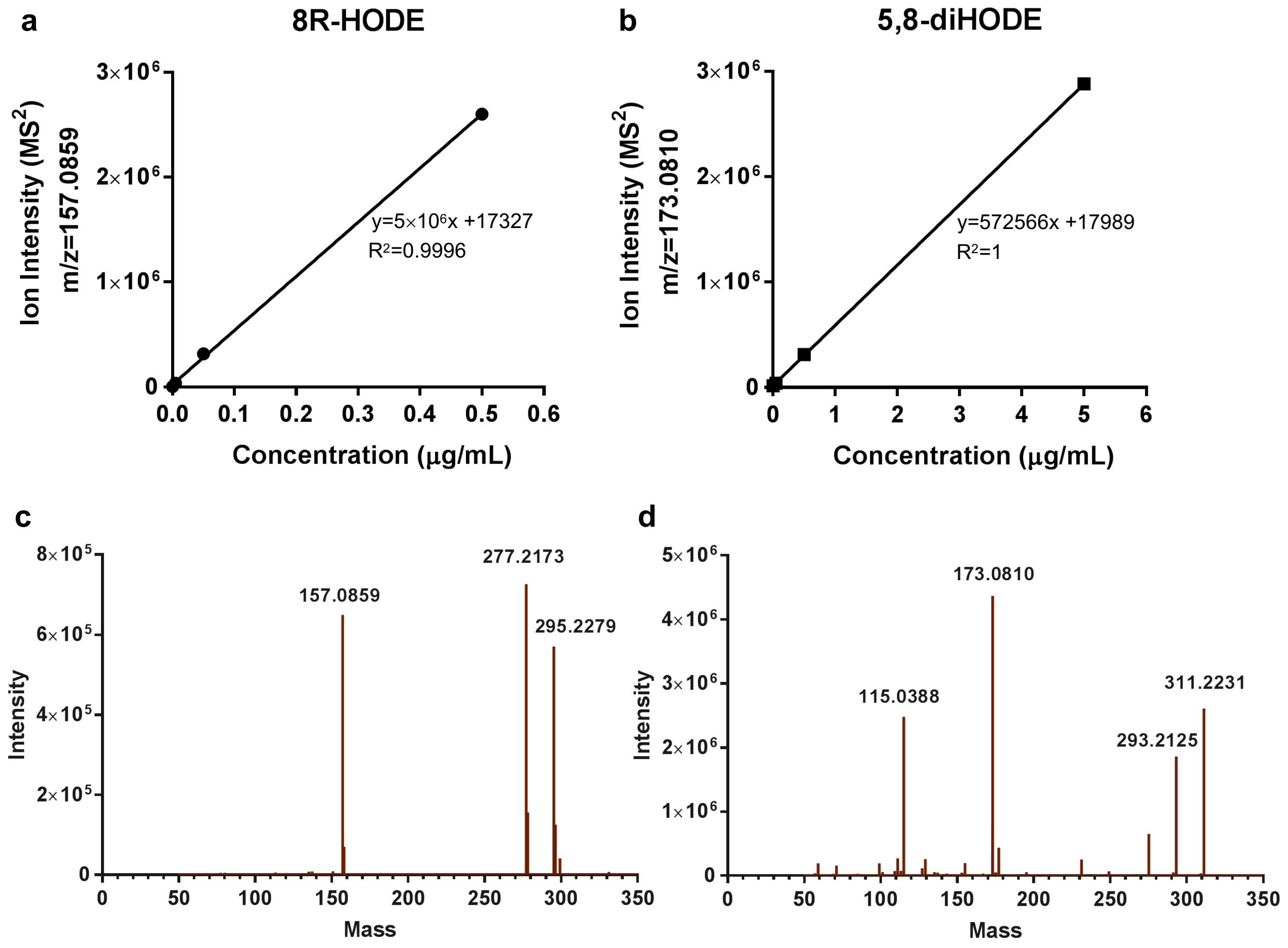

Supplementary Fig. 1. Quantification of purified PpoA oxylipins using UHPLC-MS/MS. Standard curves and linear regression equations depicting the **(a)** 8R-HODE and **(b)** 5,8-diHODE concentration *vs.* ion intensity relationship measured by UHPLC-MS/MS using purified oxylipins. Daughter $[M-H]^-$ ions with *m/z* =157.0859 and *m/z*=173.0810 were measured to quantify 8R-HODE and 5,8-diHODE abundance, respectively[19]. **c**, Tandem mass spectrum of 8R-HODE standard identifies characteristic $[M-H]^-$ ions with m/z=157.0859, 277.2173, 295.2279**; d,** Tandem mass spectrum of 5,8-diHODE standard identifies characteristic $[M-H]^-$ ions with m/z=115.0388, 173.0810, 293.2125, 311.2231**.**

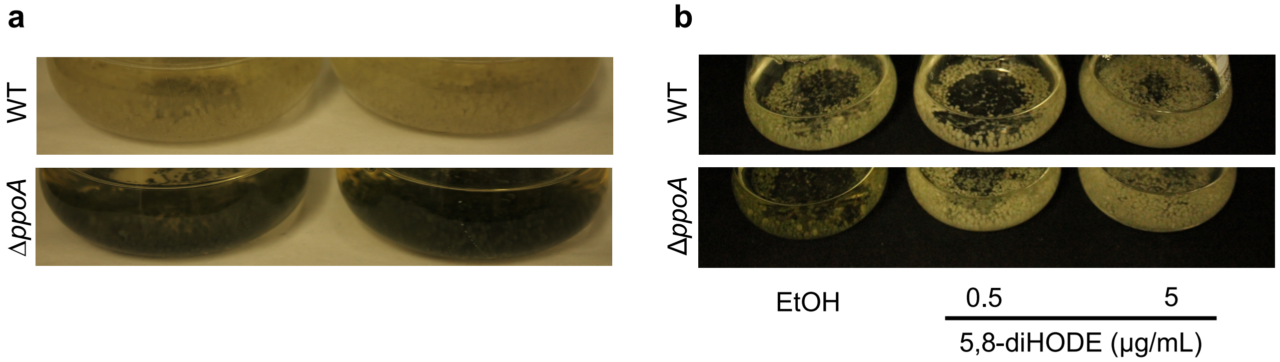

Supplementary Fig. 2. Images of *A. fumigatus* Af293 WT and Δ*ppoA* liquid cultures. a, 10^7/mL Af293 WT and Δ*ppoA* spores were inoculated in GMM and cultured at 25℃ for 5 days, under constant shaking at 250 rpm. **b,** Af293 WT and Δ*ppoA* were cultured and grown as in **(a)**, and at 65 hour-post inoculation, EtOH or 5,8-diHODE were added at the indicated concentrations. All cultures were evaluated for appearance and quantified for asexual spore production at 120 hour-post inoculation.

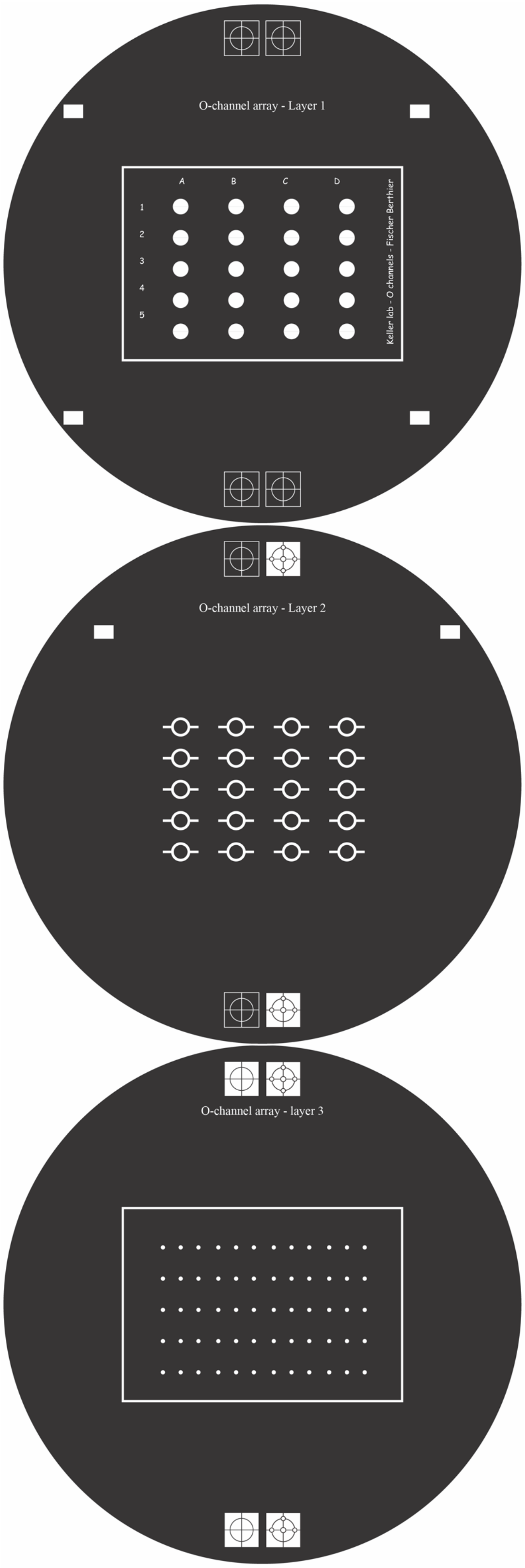

Supplementary Fig. 3. Illustration of the masters of the O-Channel microfluidic platform. A detailed fabrication method can be found in the Methods section.

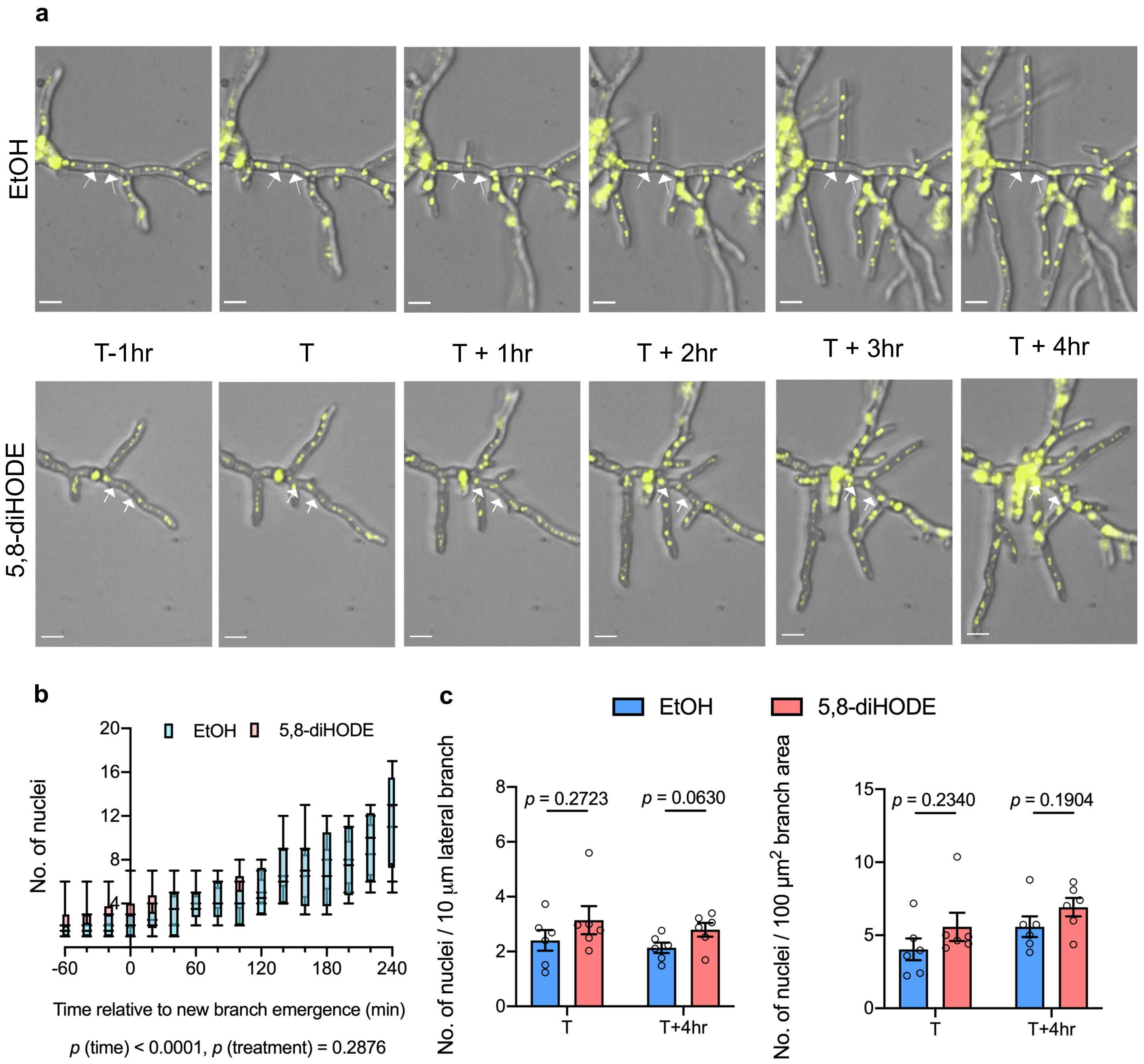
a
EtOH
5,8-diHODE
T-1hr
T
T + 1hr
T + 2hr
T + 3hr
T + 4hr
b
EtOH
5,8-diHODE
No. of nuclei
Time relative to new branch emergence (min)
p (time) < 0.0001, p (treatment) = 0.2876
c
EtOH
5,8-diHODE
No. of nuclei / 10 μm lateral branch
p = 0.2723
p = 0.0630
T
T+4hr
No. of nuclei / 100 μm² branch area
p = 0.2340
p = 0.1904
T
T+4hr

Supplementary Fig. 4. Nuclear replication at lateral branching site is not altered by 5,8-diHODE. GFP-histone tagged strain of *A. fumigatus* AF293 (TJMP 131.5) was grown for 7 hr in GMM at 37 °C before treated with 20 mM hydroxy urea for 2 hr, washed 3 times with GMM, exposed to either 1% EtOH or 5 μg/mL 5,8-diHODE, and settled for 1.5 hr briefly before time-lapse fluorescent imaging. **a,** Representative image series of EtOH and 5,8-diHODE treated hyphae. Images were cropped to focus on a cell compartment within the apical hypha, bordered by two adjacent septa as indicated in white arrows. A 5-hr window for nuclei analysis was selected based on the lateral branch emergence at time T. Images are representative of 6 microscopic images acquired for each condition. Scale bars represent 10 μm. **b**, Quantification of the number of nuclei within the apical hyphal cell compartment and the new lateral branch derived from it starting at 1 hr before branching until 4 hr after (n = 6). The box-and-whisker plots show maximum, 75% quartile, median, 25% quartile, and minimum of the data. Two-way ANOVA was performed to detect the effect of time and treatment. **c,** Nuclei counts normalized to the length (along the lateral branch direction) or area of the branch plus apical compartment. A two-way ANOVA test was performed to identify the effect of time and treatment in (**a**) and multiple two-sided t tests were used to compare normalized nuclei numbers between treatments at T+ 4hr and T in (**b**, **c**). All values represent mean ± SEM.

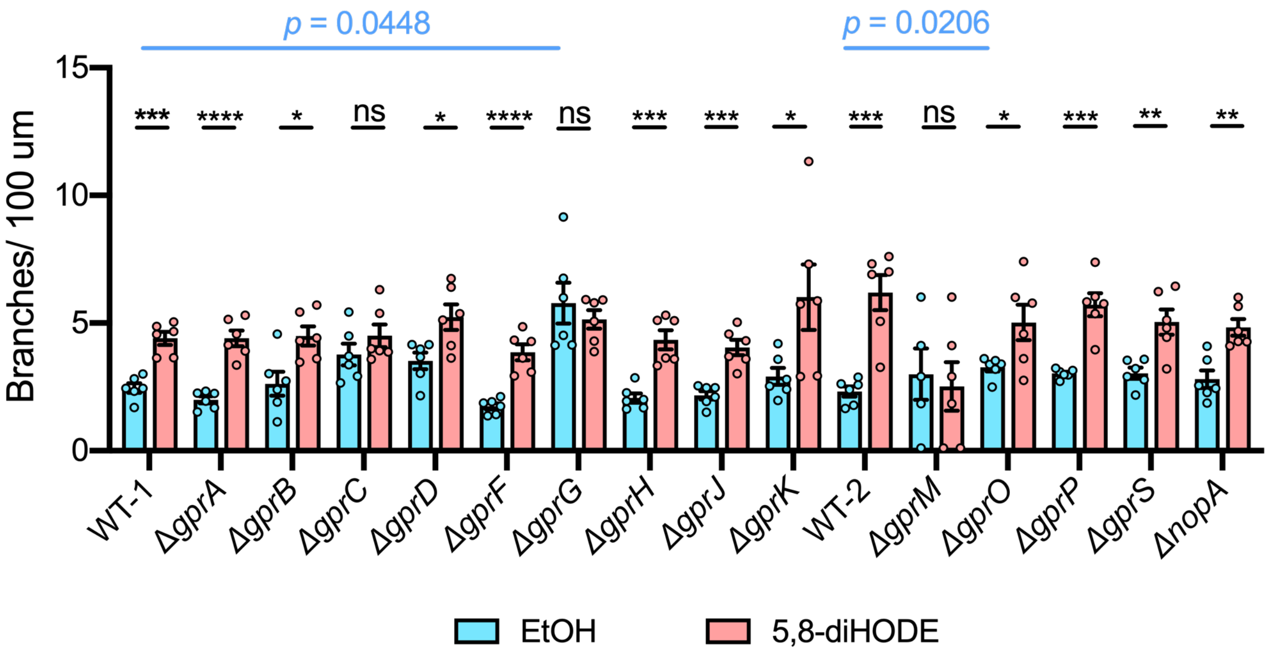

Supplementary Fig. 5. Branching assessment of *A. flavus* canonical G-protein coupled receptor (GPCR) deletion mutants identified 3 GPCRs involved in 5,8-diHODE induced branching response. *A. flavus* wildtype control (CA14 Δ*ku70*Δ*pyrG*) and GPCR mutants were cultured in microfluidic platform in GMM containing either EtOH or 5 μg/mL 5,8-diHODE. Branching was quantified at 20 hr post incubation (n = 6). 6 randomly selected hyphae from each condition in each strain were imaged and quantified for branching. Multiple two-sided t tests were performed between conditions in the same strain, while Brown-Forsythe and Welch ANOVA tests followed by Dunnett's T3 multiple comparison test were used to compare across strains within each treatment group. Values represent mean ± SEM. *P*-values corresponding to asterisks (*) from left to right are: 0.0001, <0.0001, 0.0114, 0.0169, <0.0001, 0.0003, 0.0003, 0.0410, 0.0003, 0.0343, 0.0002, 0.0038, 0.0017. ns = not significant ($p > 0.05$).

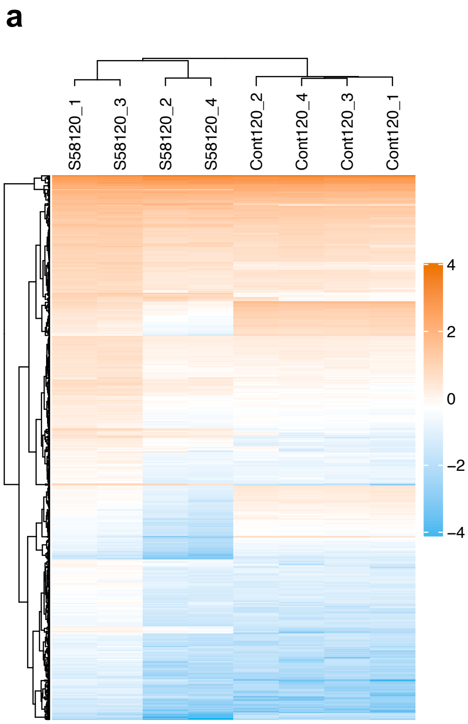

b

120 min DEGs, Up-regulated

GO Term (Molecular Function)
None

GO Term (Biological Process)
fumagillin metabolic process
fumagillin biosynthetic process
epoxide metabolic process
ether biosynthetic process
secondary metabolic process
secondary metabolite biosynthetic process
ether metabolic process
pseurotin A metabolic process
pseurotin A biosynthetic process
antibiotic biosynthetic process
antibiotic metabolic process
fumitremorgin B biosynthetic process
fumitremorgin B metabolic process
mycotoxin biosynthetic process
toxin biosynthetic process
mycotoxin metabolic process
toxin metabolic process
organic heteropentacyclic compound biosynthetic process
organic heteropentacyclic compound metabolic process
alkaloid biosynthetic process
alkaloid metabolic process
heterocycle biosynthetic process
drug metabolic process

120 min DEGs, Down-regulated

GO Term (Molecular Function)
transporter activity
transmembrane transporter activity

GO Term (Biological Process)
NA

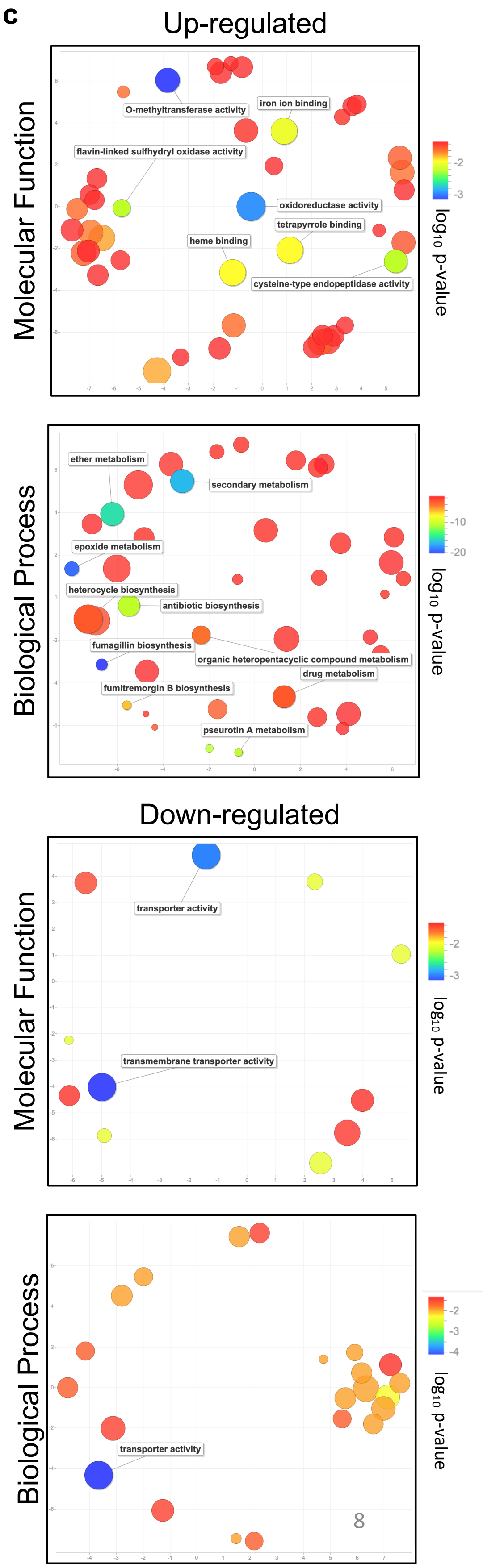

Supplementary Fig. 6. RNA sequencing analysis of the transcriptomes after 120 min exposure of hyphae to 5,8-diHODE or EtOH. a, Hierarchical clustering analysis depicts differentially expressed genes (DEGs) with FDR < 0.05 and |log2FC| > 1 at 30 min post treatment. The $\log_2$ values of FPKM (Fragments Per Kilobase of transcript per Million mapped reads) were used to construct the heat map using the ComplexHeatmap package in R. Log transformation was performed via the zfpkm package and genes with log2(FPKM)<=-3 were disregarded. Cont120: EtOH controls at 120 min; S58120: 5,8-diHODE treated samples at 120 min. **b,** GO analysis of DEGs (|Log_2FC|>1) at 120 min post treatment was performed using FungiDB. A Fisher's Exact test followed by Benjamini-Hochberg multiple-testing correction was performed and GO terms with false discovery rate < 0.05 are listed. **c,** All over-represented GO terms with Fischer's Exact test *p*-value <0.05 in the GO analysis were used to generate scatterplots with REVIGO to eliminate redundant terms. Semantic relations between GO terms are represented as the relative distance between each circle.

a

30 min DEGs, Up-regulated
**GO Term (Molecular Function)**
calcium-dependent phospholipid binding

**GO Term (Biological Process)**
fumitremorgin B biosynthetic process
fumitremorgin B metabolic process
organic heteropentacyclic compound biosynthetic process
organic heteropentacyclic compound metabolic process
indole alkaloid metabolic process
indole alkaloid biosynthetic process
alkaloid biosynthetic process
mycotoxin biosynthetic process
alkaloid metabolic process
toxin biosynthetic process
mycotoxin metabolic process
toxin metabolic process
ether biosynthetic process
verruculogen biosynthetic process
prostaglandin biosynthetic process
brevianamide F metabolic process
prostaglandin metabolic process
oxylipin metabolic process
dipeptide transport
prostanoid metabolic process
prostanoid biosynthetic process
verruculogen metabolic process
oxylipin biosynthetic process
fatty acid derivative biosynthetic process
eicosanoid metabolic process
eicosanoid biosynthetic process

30 min DEGs, Down-regulated
**GO Term (Molecular Function)**
NA
**GO Term (Biological Process)**
NA

b

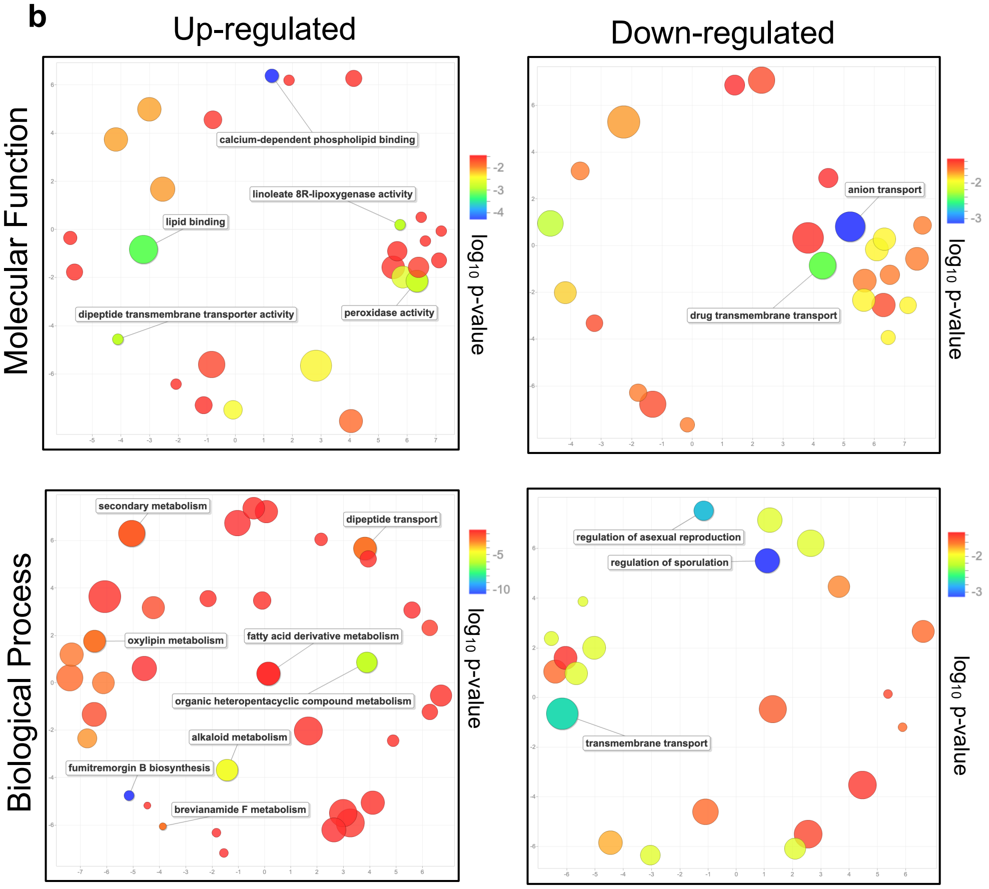

Supplementary Fig. 7. Gene Ontology (GO) analysis of over-represented differentially expressed genes with $|Log_2FC|>1$ in 5,8-diHODE-treated vs. EtOH-treated samples at 30 min post treatment. a, GO analysis of DEGs ($|Log_2FC| > 1$) at 30 min post treatment was performed using FungiDB. A Fisher's Exact test followed by Benjamini-Hochberg multiple-testing correction was performed and GO terms with false discovery rate < 0.05 are listed. **b,** All over-represented GO terms with Fischer's Exact Test *p*-value <0.05 in the GO analysis were used to generate scatterplots with REVIGO to eliminate redundant terms. Semantic relations between GO terms are represented as the relative distance between each circle.

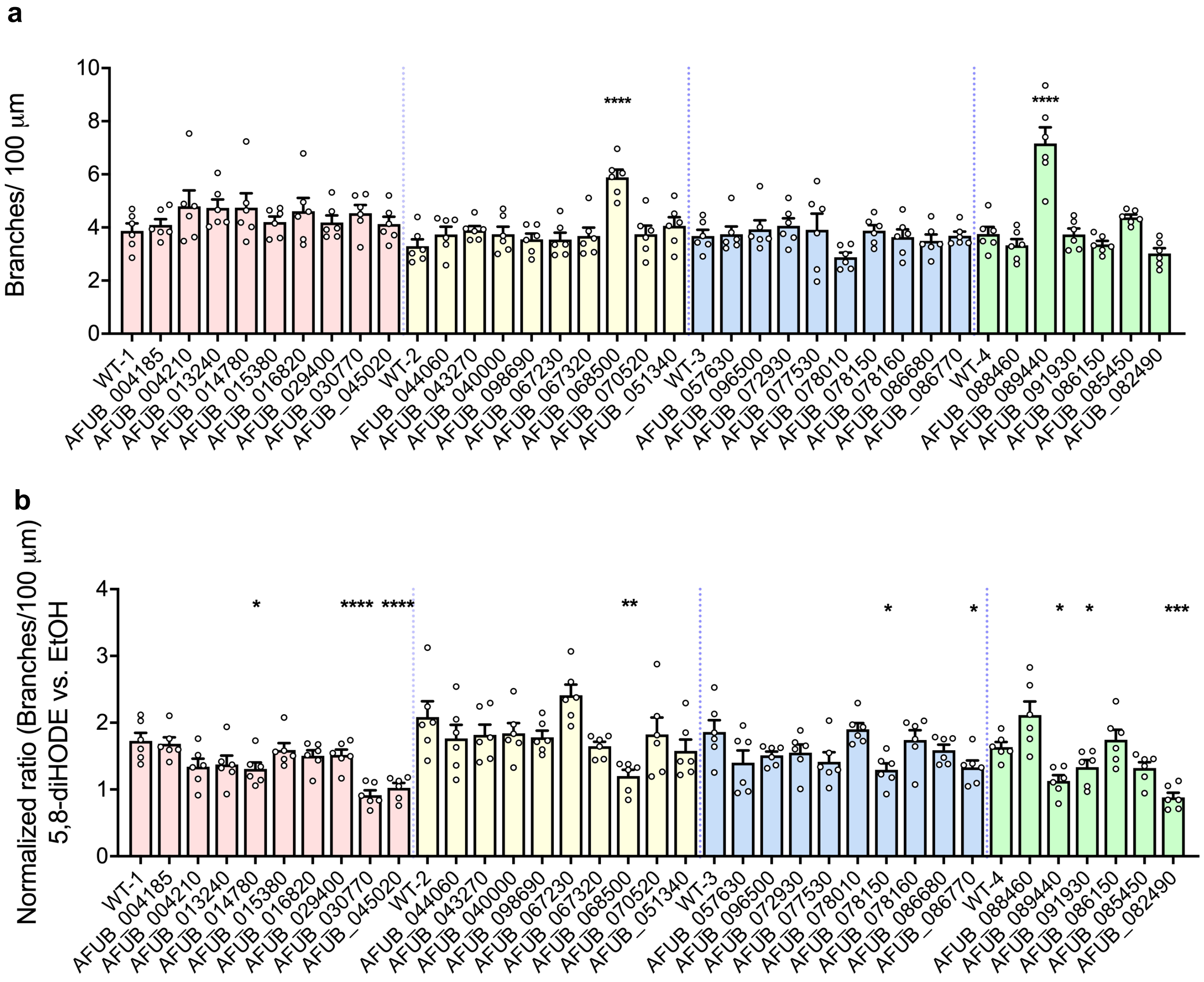

Supplementary Fig. 8. Initial screen of 33 transcription factor deletion mutants identifies 9 transcription factors showing differential hyphal branching either in GMM + 1% EtOH or in response to 5,8-diHODE (5 µg/mL). a, Branching analysis of the WT control (MFIG001) and the mutants in GMM + 1% EtOH. 1000 spores in 100 µL were inoculated in 96-well plate, incubated for 15 hr before time-lapse imaging. Hyphal branching analysis was performed on images acquired at 20 hr post incubation. Strains with the same color were screened in the same batch and compared to the WT in that batch. *P*-values corresponding to asterisks (*) from left to right are: <0.0001, <0.0001. **b,** Normalized ratios of branches/100 µm of all strains grown in GMM + 5,8-diHODE vs. GMM + EtOH. *P*-values corresponding to asterisks (*) from left to right are: 0.0321, <0.0001, <0.0001, 0.0044, 0.0184, 0.0313, 0.0388, 0.0255, 0.0005. Ordinary one-way ANOVA with post-hoc Dunnett's multiple comparison test was used to compare between mutant and the corresponding WT control. Data obtained from different experiments are separated by vertical dashed lines. All values represent mean ± SEM.

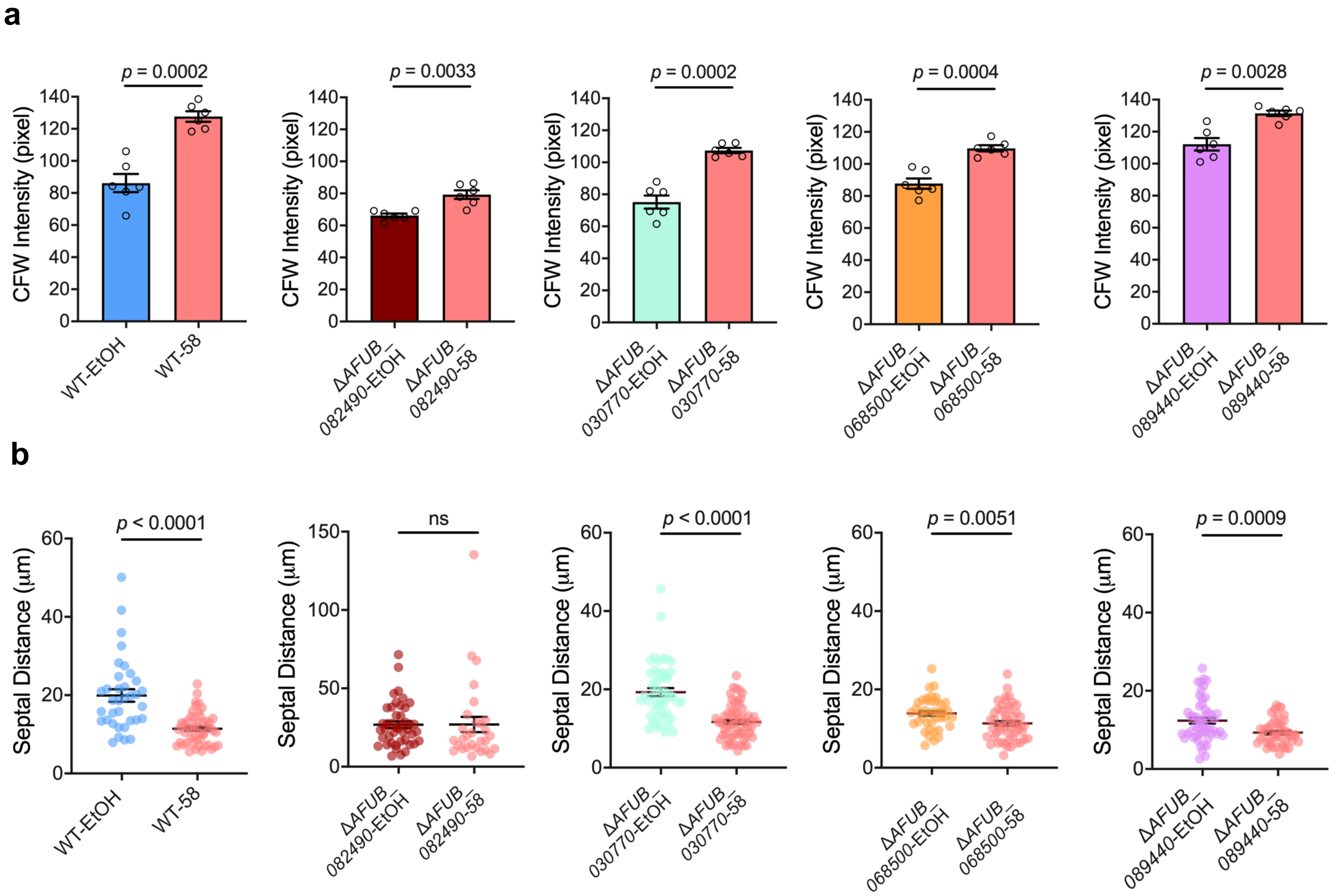

Supplementary Fig. 9*. Deletion mutants of transcription factors display alterations in septal distance and chitin content in hyphae.* a, Quantification of CFW fluorescence intensity in CEA10 TF mutants and the A1160 *pyrG*$^{+}$ WT control. 2500 spores in 400 μL were inoculated in 24-well plate pre-mounted with coverslip at the bottom. All cultures were incubated for 14 hr before CFW staining and imaging. Acquired DAPI images were analyzed in FIJI. Hyphae in each image was selected through the thresholding function in FIJI, and the mean fluorescence intensity of each threshold area was measured. **b,** Quantification of septal distance in CEA10 TF mutants and the A1160 *pyrG*$^{+}$ WT control. Septa were visualized in the acquired DAPI images and distances were measured in the NIS software package. All comparisons were made using two-sided Welch's t-test. All values represent mean ± SEM. ns = not significant ($p > 0.05$).

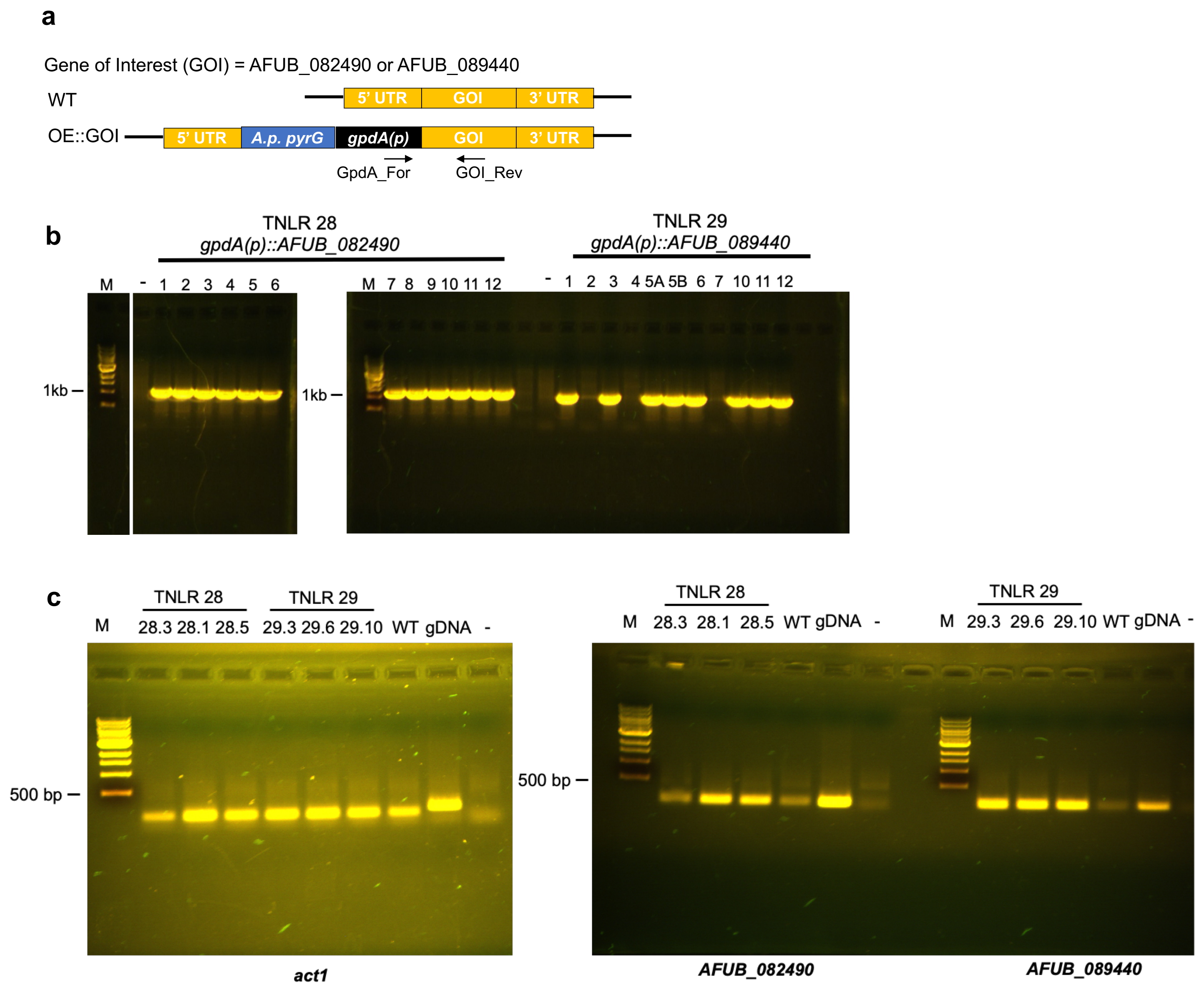

Supplementary Fig. 10. Construction of the *AFUB_082490* and *AFUB_089440* overexpression mutants. a, Genetic contexts of the wildtype (A1160 *pyrG*$^+$) and the OE::*AFUB_082490.* Double fusion PCR was performed to create transformation genetic construct to insert the *A. nidulans gpdA* promoter fused with the selection marker *A. parasiticus pyrG* in front of the transcription start site of *AFUB_082490 or AFUB_089440.* The genetic construct was transformed into uridine/uracil auxotroph CEA17 Δ*pyrG.* Primer pairs of the forward primer gpdA_For and the reverse primer GOI_Rev were used for positive selection of target mutants via PCR. **b,** Results of PCR screen of transformation mutants. A 1kb fragment was expected to be amplified from correct OE::*AFUB_082490* TNLR 28 an OE::*AFUB_089440* TNLR 29 mutants. **c,** Assessment of *AFUB_ 082490* and *AFUB_089440* expression in 3 independent transformants of TNLR 28 and TNLR 29 confirmed in **(b)** and the WT A1160 *pyrG*$^+$ through semi-quantitative PCR. *act1* was assessed visually to control for even loading. gDNA and water were used as positive and negative control for PCR.

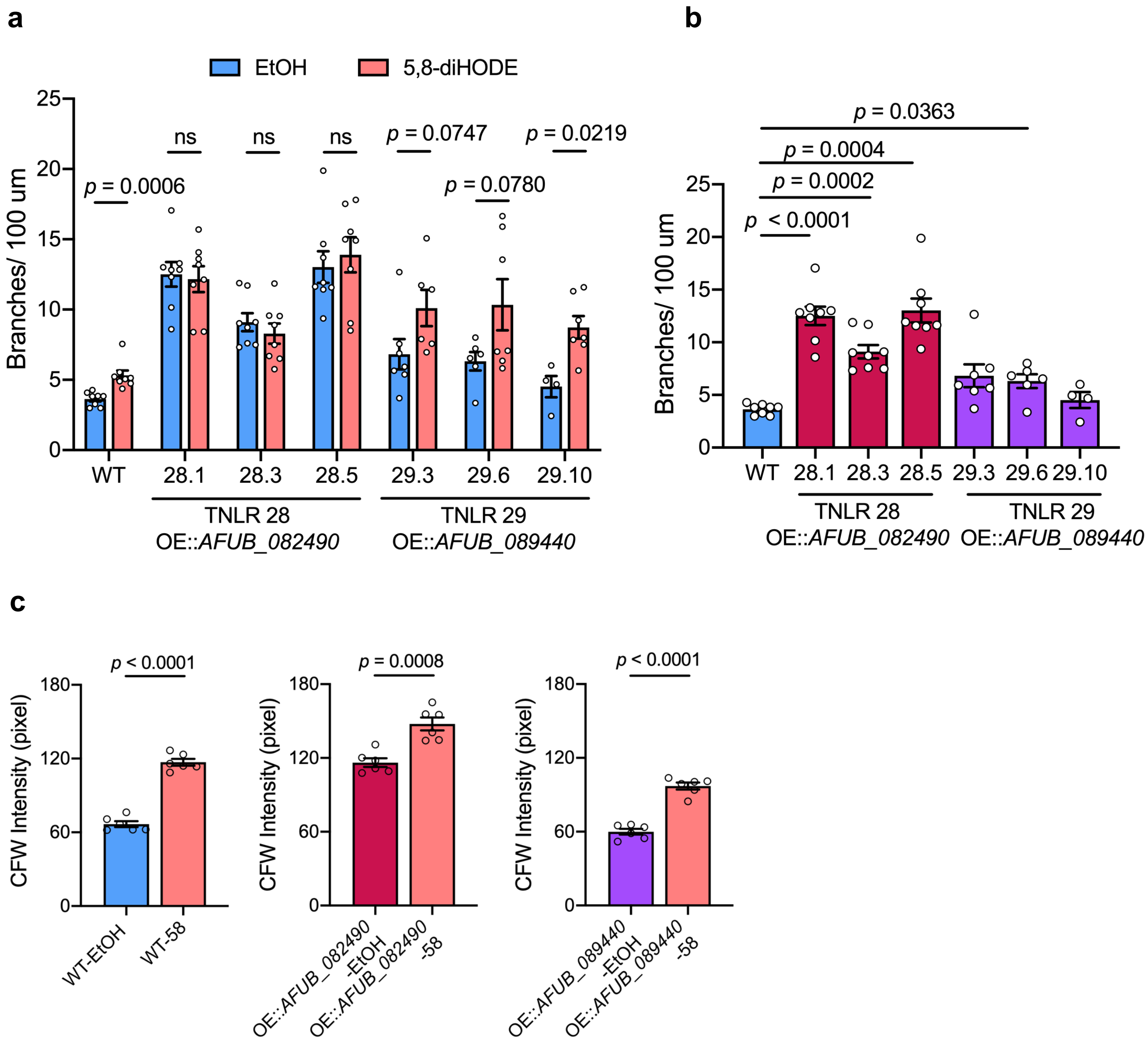

Supplementary Fig. 11. Phenotype assessment of OE::*AFUB_082490* and OE::*AFUB_089440* mutants. a, Assessment of branching response of the A1160 *pyrG*+ WT control and 3 independent transformants of OE::*AFUB_082490* and OE::*AFUB_089440*. Branching assessment was performed in 96-well format with hyphae grown in GMM + 1% EtOH or GMM + 5 µg/mL of 5,8-diHODE. Quantification was performed on 20 hr-old hyphae that contained more than 2 lateral branches at time of data acquisition (n = 8 for WT and TNR 28 mutants, n = 7 for TNR 29.3, n = 6 for TNR 29.6, and n = 4 for TNR 29.10). **b,** Branching levels of the A1160 *pyrG*+ WT, OE::*AFUB_082490*, and OE::*AFUB_089440* when grown in GMM + 1% EtOH in **(a)**. **c,** CFW intensity of hyphae from the A1160 *pyrG*+ WT and the TF overexpression strains, quantified using images acquired in **Fig. 7a** as described in the Methods section. Two-sided Welch's t-test was used to compare between treatment groups in (**a, c**) and Brown-Forsythe and Welch ANOVA tests followed by post-hoc Dunnett's T3 multiple comparison test were used to compare across strains in (**b**). All values represent mean ± SEM. ns = not significant ($p > 0.05$).

a

Gene ID	Log_2FC (30 min)	Log_2FC (120 min)	Gene Description
AFUA_1G03800	0.97884	1.1427	C6 transcription factor, putative
AFUA_1G13750	0.84172	1.3687	C2H2 transcription factor (Rpn4), putative
AFUA_1G15230	0.71812	1.0266	C6 transcription factor, putative
AFUA_1G15850	0.82339	1.1581	C6 transcription factor, putative
AFUA_1G17460	1.4327	1.1581	C6 transcription factor, putative
AFUA_3G02590	1.0865	1.1647	C6 transcription factor, putative
AFUA_3G03230	0.49477	1.1139	bZIP transcription factor, putative
AFUA_3G03900	1.2672	1.2637	C6 transcription factor, putative
AFUA_3G05760	1.2526	1.0585	C6 transcription factor (Fcr1), putative
AFUA_3G09130	1.4708	0.75138	C6 transcription factor, putative
AFUA_4G10110	0.8675	1.0677	homeobox transcription factor, putative
AFUA_4G10200	1.1313	1.2302	transcription factor RfeF, putative
AFUA_5G10040	-	1.1284	C6 transcription factor, putative
AFUA_6G01840	1.0192	0.71543	C6 transcription factor, putative
AFUA_6G07010	-	1.7052	C6 transcription factor RosA
AFUA_6G11520	2.4579	1.4945	C6 transcription factor, putative
AFUA_6G12020	1.1448	-	C2H2 finger domain protein, putative
AFUA_6G12150	1.4053	-	bZIP transcription factor (Atf7), putative
AFUA_6G12160	1.4865	1.2913	C6 transcription factor, putative
AFUA_7G00210	1.0245	1.0559	C6 transcription factor, putative
AFUA_7G03910	1.0648	1.2933	C2H2 zinc finger protein
AFUA_7G04820	-	1.7195	C6 transcription factor, putative
AFUA_8G00420	1.0539	1.4364	C6 finger transcription factor, putative
AFUA_8G05010	2.6181	3.4992	C2H2 finger domain protein, putative

b

Gene ID	Log_2FC (30 min)	Log_2FC (120 min)	Gene Description
AFUA_1G03780	-1.1048	-0.63548	C6 finger domain protein, putative
AFUA_2G13770	-1.0264	-	C2H2 conidiation transcription factor FlbC
AFUA_2G15110	-2.2696	-	C2H2 finger domain protein, putative
AFUA_4G04320	-1.2774	-2.0421	homeobox transcription factor, putative
AFUA_4G11480	-1.4949	-	C2H2 finger domain protein, putative
AFUA_4G13600	-1.3444	-	C2H2 finger domain protein, putative
AFUA_5G02800	-1.1179	-0.75052	C6 transcription factor, putative
AFUA_7G00130	-1.3014	-	C6 transcription factor, putative
AFUA_7G01890	-0.69089	-1.2652	C6 transcription factor, putative
AFUA_7G06370	-1.1062	-	C6 transcription factor, putative
AFUA_8G01150	-0.96336	-1.0155	C6 transcription factor, putative

Supplementary Table 1. List of differentially expressed genes (DEGs) that encode putative transcriptional factors and have more than 2-fold **(a)** under- or **(b)** over-expression when hyphae were treated with 5 μg/mL 5,8-diHODE compared with EtOH control in the RNA Sequencing experiment. Yellow cells represent DEGs at both 30 min and 120 min, blue cells represent DEGs at 30 min only, purple cells represent DEGs at 120 min only.

Name	Sequence (5’-3’)	Purpose
MN_AFUA_2G15110_FOR	ATGCATTCAAGCGCTTATGCC	qRT-PCR of AFUA_2G15110 Forward
MN_AFUA_2G15110_REV	GGACGATCGCAGTTGAAATTGAG	qRT-PCR of AFUA_2G15110 Reverse
MN_act1_FOR	CTTCCAGCCTAGCGTTCT	qRT-PCR of actin1_Forward
MN_act1_REV	GTACATGGTGGTACCACCAG	qRT-PCR of actin1_Reverse
MN_aslA_FOR	CGTAAATCCGATCTCTGCAGAC	qRT-PCR of AFUA_4G11480 Forward
MN_aslA_REV	GAGGCTTTTCGCCAGTGTG	qRT-PCR of AFUA_4G11480 Forward
MN_nsdC_FOR	GGCTGCATTGACGCAATCGAC	qRT-PCR of AFUA_7G03910 Forward
MN_nsdC_REV	CGATGGACGTCAAGCTCGAATC	qRT-PCR of AFUA_7G03910 Reverse
MN_zfpA_FOR	GGACTCGTCACGTCAACAAC	qRT-PCR of AFUA_8G05010 Forward
MN_zfpA_REV	CCGTAATAAGATGTTGGTGCGC	qRT-PCR of AFUA_8G05010 Reverse
NR AFUB_082490 5' FOR	TTATTTTGCCCGACTCTTGG	Creating OE::AFUB_082490 strain
NR AFUB_082490 NEST FOR	CAATGCACACCAGATGATCC	Creating OE::AFUB_082490 strain
NR AFUB_082490 5' REV	CCCTATAGTGAGTCGTATTACGG ATGGCTTCTTTGTGCGCTAAT	Creating OE::AFUB_082490 strain
NR AFUB_082490 3' FOR	CTTGAGCAGACATCACCATGATG TTGGCATTGGACTCGTC	Creating OE::AFUB_082490 strain
NR AFUB_082490 NEST REV	TTCCCTTGTAATGGGTGAGC	Creating OE::AFUB_082490 strain
NR AFUB_082490 3' REV	CGCTTAAAGACATCCGCTCT	Creating OE::AFUB_082490 strain
NR AFUB_089440 5' FOR	GTGTTCGCCCTCAGAAAGAG	Creating OE::AFUB_089440 strain
NR AFUB_089440 NEST FOR	GCCCATCAGTCAGTCCATCT	Creating OE::AFUB_089440 strain
NR AFUB_089440 5' REV	CCCTATAGTGAGTCGTATTACGG GATAACAGATGCTTGTTGTCCGC C	Creating OE::AFUB_089440 strain
NR AFUB_089440 3' FOR	CTTGAGCAGACATCACCATGATG GCTGCATTGACGCAATC	Creating OE::AFUB_089440 strain
NR AFUB_089440 NEST REV	GCGCAAAACTTCCCATACTG	Creating OE::AFUB_089440 strain
NR AFUB_089440 3' REV	GACCGTCCATGGTATCTTCG	Creating OE::AFUB_089440 strain
NLR_gpdA_FOR	ATTCATCTTCCCATCCAAGAACC	PCR screen of OE::TF transformants_Forward
NLR_zfpA_REV	CGCTTAAAGACATCCGCTCT	PCR screen for TNLR 28 transformants_Reverse
NLR_nsdC_REV	GACCGTCCATGGTATCTTCG	PCR screen for TNLR 29 transformants_Reverse

Supplementary Table 2. Primers used in this study.
