# Supplementary material for: Fungal oxylipins direct programmed developmental switches in filamentous fungi
Source: Nat Commun. 2020 Oct 14;11:5158. doi: 10.1038/s41467-020-18999-0 (PMC7557911; doi:10.1038/s41467-020-18999-0)
Supplement: Supplementary file 3 — Description of Additional Supplementary Files [file 41467_2020_18999_MOESM3_ESM.pdf]

Description of Additional Supplementary Files

File Name: Supplementary Data 1
Description: Differentially expressed genes (DEGs) identified in the RNA Sequencing experiment. All DEGs with *P*-value < 0.05 are listed with *P*-values designated for each gene.

File Name: Supplementary Data 2
Description: Gene Ontology (GO) terms of over-represented by differentially expressed genes with Log_2(fold change) >1 at 30 min and 120 min in the RNA Sequencing experiment. A Fisher's Exact test followed by Benjamini-Hochberg multiple-testing correction was performed and GO terms with false discovery rate < 0.05 are listed.

File Name: Supplementary Movie 1
Description: Time-lapse images of germinated Af293 growing in GMM containing EtOH at 37 15-20 hours post incubation. Scale bar represents 50 µm.

File Name: Supplementary Movie 2
Description: Time-lapse images of germinated Af293 growing in GMM containing 5 µg/mL 5,8-diHODE at 15-20 hours post incubation. Scale bar represents 50 µm.

File Name: Supplementary Movie 3
Description: Time-lapse images of germinated CEA10 growing in GMM containing EtOH at 15-20 hours post incubation. Scale bar represents 50 µm.

File Name: Supplementary Movie 4
Description: Time-lapse images of germinated CEA10 growing in GMM containing 5 µg/mL 5,8-diHODE at 15-20 hours post incubation. Scale bar represents 50 µm.

File Name: Supplementary Movie 5
Description: Time-lapse images of germinated Δ*AFUB_089440/nsdC* growing in GMM containing EtOH at 19-23.5 hour-post incubation. Scale bar represents 50 µm.
