# Supplementary material for: Fungal oxylipins direct programmed developmental switches in filamentous fungi
Source: Nat Commun. 2020 Oct 14;11:5158. doi: 10.1038/s41467-020-18999-0 (PMC7557911; doi:10.1038/s41467-020-18999-0)
Supplement: Supplementary file 11 — Reporting Summary [file 41467_2020_18999_MOESM11_ESM.pdf]

nature research

Corresponding author(s): Nancy P. Keller

Last updated by author(s): Aug 23, 2020

Reporting Summary

Nature Research wishes to improve the reproducibility of the work that we publish. This form provides structure for consistency and transparency in reporting. For further information on Nature Research policies, see Authors & Referees and the Editorial Policy Checklist .

Statistics

For all statistical analyses, confirm that the following items are present in the figure legend, table legend, main text, or Methods section.

n/a	Confirmed	
	x	The exact sample size (*n*) for each experimental group/condition, given as a discrete number and unit of measurement
	x	A statement on whether measurements were taken from distinct samples or whether the same sample was measured repeatedly
	x	The statistical test(s) used AND whether they are one- or two-sided<br>*Only common tests should be described solely by name; describe more complex techniques in the Methods section.*
x		A description of all covariates tested
	x	A description of any assumptions or corrections, such as tests of normality and adjustment for multiple comparisons
	x	A full description of the statistical parameters including central tendency (e.g. means) or other basic estimates (e.g. regression coefficient) AND variation (e.g. standard deviation) or associated estimates of uncertainty (e.g. confidence intervals)
	x	For null hypothesis testing, the test statistic (e.g. *F*, *t*, *r*) with confidence intervals, effect sizes, degrees of freedom and *P* value noted<br>*Give P values as exact values whenever suitable.*
x		For Bayesian analysis, information on the choice of priors and Markov chain Monte Carlo settings
	x	For hierarchical and complex designs, identification of the appropriate level for tests and full reporting of outcomes
x		Estimates of effect sizes (e.g. Cohen's *d*, Pearson's *r*), indicating how they were calculated

Our web collection on statistics for biologists contains articles on many of the points above.

Software and code

Policy information about availability of computer code

Data collection	Transcriptomes were collected using the Illumina Hiseq2500 system and library was prepared. Reads were mapped in reference to genome of Aspergillus fumigatus Af293 (release 40) using TopHat (v2.0.12). Oxylipin quantification was performed using the Thermo Xcalibur Qual Browser (v. 3.1.66.10). Quantification of hyphal branching, septal distance and nuclear division was performed in the Nikon NIS Elements AR software package (Version 4.13). Fluorescence quantification was performed in FIJI (Version 2.0.0-rc-69/1.52p).
Data analysis	In RNA sequencing analysis, mapped reads were analyzed for differential gene expression using DESeq2 (v1.10.1) and HTSeq (v0.6.1) to calculate Fragments Per Kilobase of transcript per Millions mapped reads (FPKM). Heat maps were drawn using the R packages zFPKM (v. 1.8.0) for log transformation and ComplexHeatmap (v. 2.2.0) in RStudio (Version 1.1.463). Gene Ontology of differentially expressed genes (FDR<0.05 and |log2 fold change| >1) was analyzed for enrichment in FungiDB53 (https://fungidb.org/fungidb/) and visualized as scatter plots in REVIGO.

For manuscripts utilizing custom algorithms or software that are central to the research but not yet described in published literature, software must be made available to editors/reviewers. We strongly encourage code deposition in a community repository (e.g. GitHub). See the Nature Research guidelines for submitting code & software for further information.

Data

Policy information about availability of data

All manuscripts must include a data availability statement. This statement should provide the following information, where applicable:

- Accession codes, unique identifiers, or web links for publicly available datasets
- A list of figures that have associated raw data
- A description of any restrictions on data availability

The methods of data collection and analysis are included in the methods section. The list of differentially expressed genes from the RNA sequencing experiment is provided in Supplementary Data 1. FungiDB (https://fungidb.org/fungidb/) was used for access to A. fumigatus Af293 genome (release 40). RNA sequencing data supporting the findings in this study has been deposited to the NCBI Gene Expression Omnibus with the identifier GSE156537 [https://www.ncbi.nlm.nih.gov/geo/query/acc.cgi?acc=GSE156537]. All data obtained to support the findings of this study are available within the article and its supplementary materials, or from the

corresponding author upon request.

Field-specific reporting

Please select the one below that is the best fit for your research. If you are not sure, read the appropriate sections before making your selection.

- [x] Life sciences
- [ ] Behavioural & social sciences
- [ ] Ecological, evolutionary & environmental sciences

For a reference copy of the document with all sections, see nature.com/documents/nr-reporting-summary-flat.pdf

Life sciences study design

All studies must disclose on these points even when the disclosure is negative.

Sample size	Initial sample size was determined through pilot tests where 5-6 replicates robustly identified differential hyphal branching between treatment and control groups. Later studies increased sample size to 8 in well-plate to screen genetic mutants in CEA17, another wildtype strain background of Aspergillus fumigatus, as this strain grew at a faster rate and imaging for branching quantification is slightly more challenging. The sample size of RNA sequencing experiment was determined considering the covariates contained within the transcriptome and the impact of 5,8-diHODE on fungal branching.
Data exclusions	No data was excluded.
Replication	Each figure, as explained in the text, consisted of 3-8 replications. Furthermore, branching assessments were performed repetitively using separate batches of purified 5,8-diHODE which consistently gave the same biological results. Fungal genetic mutant screening and confirmation were performed in a similar manner across different platforms and setups (e.g. microfluidic device, well-plates, growth on cover-slip), all which gave the same biological results.
Randomization	Treatment and control groups were always inoculated into the same well-plates or microfluidic devices in a randomized design.
Blinding	The researcher who analyzed the initial branching screening using all 33 transcription factors was blinded for the location of treatment vs. control samples. Two different people analyzed the experiments and obtained the same results.

Reporting for specific materials, systems and methods

We require information from authors about some types of materials, experimental systems and methods used in many studies. Here, indicate whether each material, system or method listed is relevant to your study. If you are not sure if a list item applies to your research, read the appropriate section before selecting a response.

Materials & experimental systems

n/a	Involved in the study	
[x]	[ ]	Antibodies
[x]	[ ]	Eukaryotic cell lines
[x]	[ ]	Palaeontology
[x]	[ ]	Animals and other organisms
[x]	[ ]	Human research participants
[x]	[ ]	Clinical data

Methods

n/a	Involved in the study	
[x]	[ ]	ChIP-seq
[x]	[ ]	Flow cytometry
[x]	[ ]	MRI-based neuroimaging
